# Supplementary material for: Proteome-wide Mendelian randomization in global biobank meta-analysis reveals multi-ancestry drug targets for common diseases
Source: Cell Genom. 2022 Oct 12;2(11):100195. doi: 10.1016/j.xgen.2022.100195 (PMC9646482; doi:10.1016/j.xgen.2022.100195)
Supplement: Document S1. Figures S1–S3 and Tables S6 and S20 [file mmc1.pdf]

Cell Genomics, Volume *2*

Supplemental information

Proteome-wide Mendelian randomization in global biobank meta-analysis reveals multi-ancestry drug targets for common diseases

Huiling Zhao, Humaria Rasheed, Therese Haugdahl Nøst, Yoonsu Cho, Yi Liu, Laxmi Bhatta, Arjun Bhattacharya, Global Biobank Meta-analysis Initiative, Gibran Hemani, George Davey Smith, Ben Michael Brumpton, Wei Zhou, Benjamin M. Neale, Tom R. Gaunt, and Jie Zheng

Supplementary materials

Proteome-wide Mendelian randomization in global biobank meta-analysis reveals multi-ancestry drug targets for common diseases

Huiling Zhao, Humaria Rasheed, Therese Haugdahl Nøst, Yoonsu Cho, Yi Liu, Laxmi Bhatta, Arjun Bhattacharya, Global Biobank Meta-analysis Initiative, Gibran Hemani, George Davey Smith, Ben Michael Brumpton, Wei Zhou, Benjamin M. Neale, Tom R. Gaunt, Jie Zheng

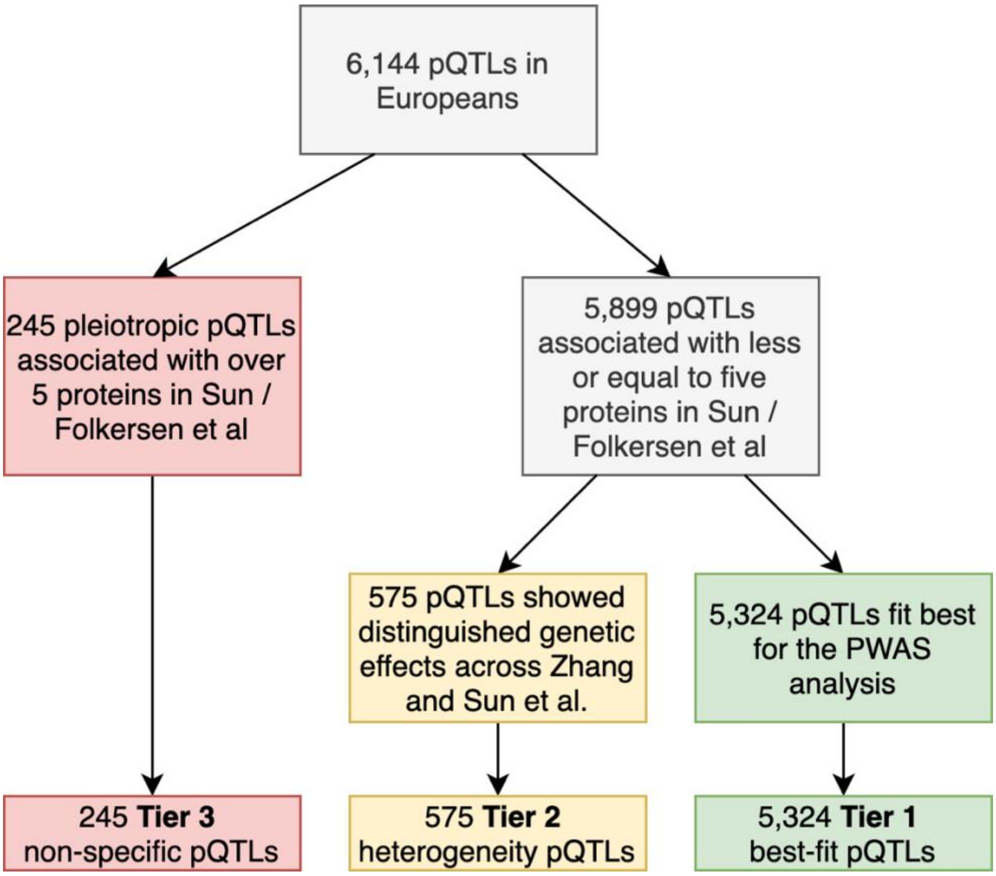

Figure S1. Instrument validation using a tier system, related to STAR method

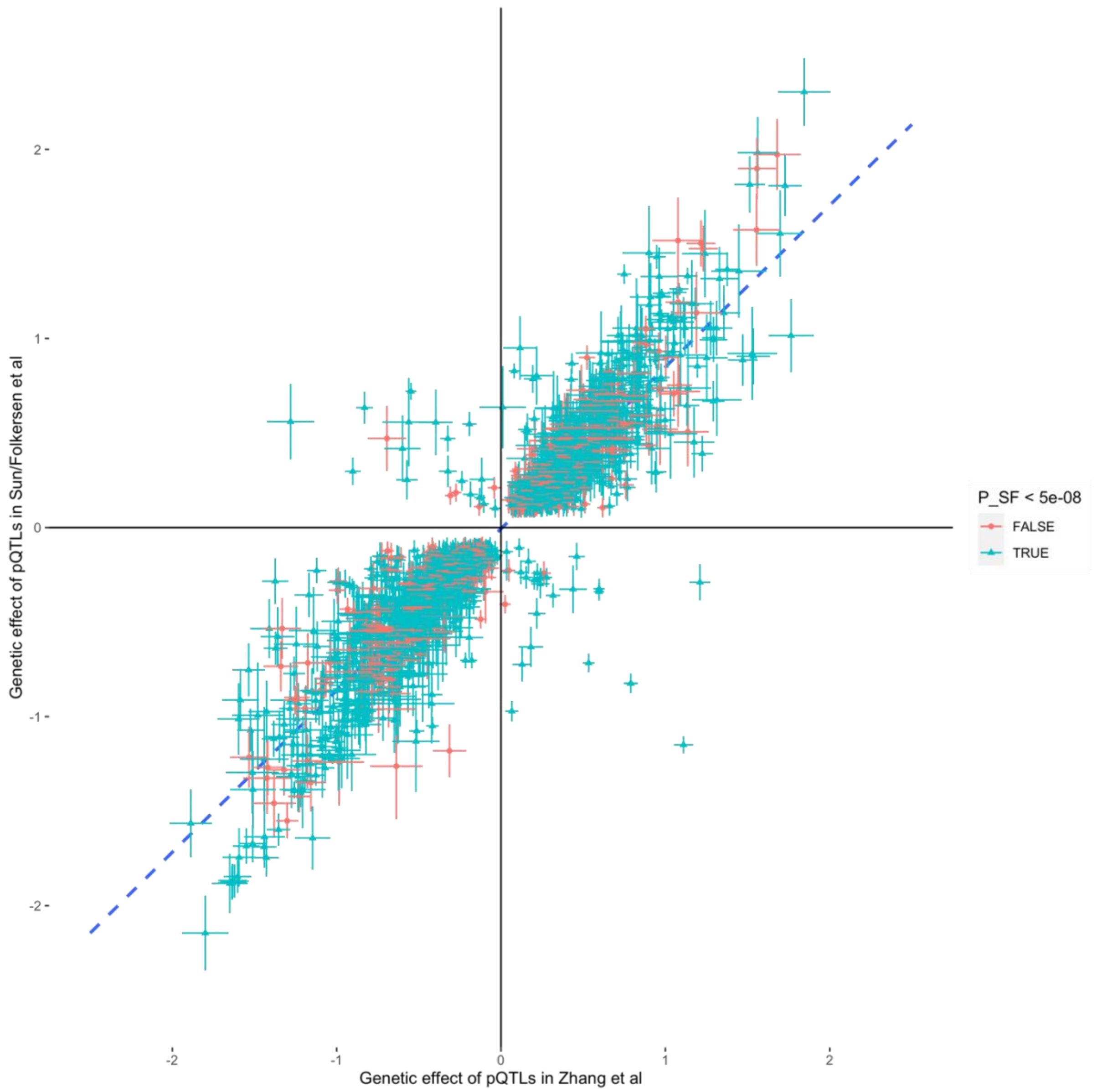

Figure S2. Comparing genetic effects of pQTLs across Zhang et al and Sun / Folkersen et al. Pearson correlation = 0.92, related to STAR method

Protein target	Disease	N SNPs	N cases/N total	Odds ratio(95%CI)	P value
SERPINE2	COPD	6	51644/802279	0.97(0.96,0.99)	9.28e-05
SERPINE2	VTE	6	15970/697076	0.94(0.92,0.96)	7.31e-08
SERPINE2	IPF	6	5229/755859	0.95(0.91,0.99)	0.0099

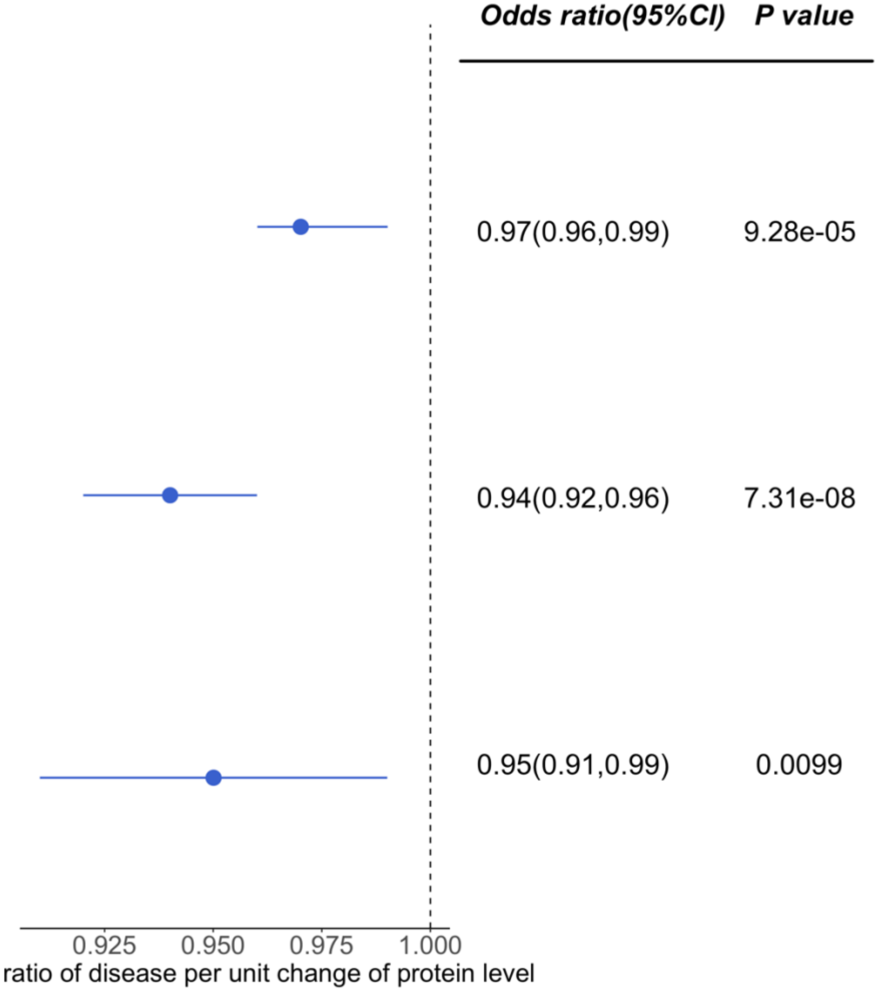

Figure S3. The putative causal effect of protein level of SERPINE2 on three tested diseases, related to STAR method

Table S6A. Information of the GBMI African-specific GWASs, related to STAR method

Endpoint	name	Sex	Dataset	Biobank	Ancestry	Case	Control	N_total	N_SNPs
Idiopathic pulmonary fibrosis	IPF	Bothsex	Total	2	1	169	8,368	8,537	18,058,634
Primary open-angle glaucoma	POAG	Bothsex	Total	3	1	483	26,323	26,806	15,534,506
Heart failure	HF	Bothsex	Total	4	1	1,367	29,835	31,202	24,631,161
Venous thromboembolism	VTE	Bothsex	Total	4	1	1,466	31,042	32,508	24,935,323
Stroke	Stroke	Bothsex	Total	4	1	1,161	24,416	25,577	21,994,124
Gout	Gout	Bothsex	Total	6	1	1,312	33,902	35,214	25,056,505
Chronic obstructive pulmonary disease	COPD	Bothsex	Total	6	1	1,978	27,704	29,682	23,889,093
Asthma	Asthma	Bothsex	Total	6	1	5,051	27,607	32,658	24,660,533
Idiopathic pulmonary fibrosis	IPF	Female	Total	NR	NR	NR	NR	NR	NR
Primary open-angle glaucoma	POAG	Female	Total	2	1	249	12,361	12,610	15,313,506
Heart failure	HF	Female	Total	2	1	542	12,211	12,753	16,366,512
Venous thromboembolism	VTE	Female	Total	4	1	795	18,306	19,101	22,266,615
Stroke	Stroke	Female	Total	3	1	639	14,459	15,098	19,332,406
Gout	Gout	Female	Total	2	1	405	13,731	14,136	17,064,891
Chronic obstructive pulmonary disease	COPD	Female	Total	5	1	1,060	15,990	17,050	21,218,274
Asthma	Asthma	Female	Total	5	1	3,408	15,993	19,401	22,046,242
Idiopathic pulmonary fibrosis	IPF	Male	Total	NR	NR	NR	NR	NR	NR
Primary open-angle glaucoma	POAG	Male	Total	2	1	155	7,705	7,860	14,154,062
Heart failure	HF	Male	Total	3	1	466	10,006	10,472	19,265,630
Venous thromboembolism	VTE	Male	Total	2	1	18,392	276,146	294,538	19,167,224
Stroke	Stroke	Male	Total	2	1	347	7,737	8,084	14,526,945
Gout	Gout	Male	Total	4	1	648	9,713	10,361	17,289,485
Chronic obstructive pulmonary disease	COPD	Male	Total	4	1	692	10,385	11,077	19,835,727
Asthma	Asthma	Male	Total	5	1	1,362	10,733	12,095	20,105,752

Table S6B. Information of the GBMI European-specific GWASs, related to STAR method

Endpoint	name	Sex	Dataset	Biobank	Ancestry	Case	Control	N_total	N_SNPs
Idiopathic pulmonary fibrosis	IPF	Bothsex	Total	8	1	5,229	750,630	755,859	45,621,626
Primary open-angle glaucoma	POAG	Bothsex	Total	11	1	11,922	946,349	958,271	46,421,873
Heart failure	HF	Bothsex	Total	9	1	28,795	772,854	801,649	46,480,843
Venous thromboembolism	VTE	Bothsex	Total	6	1	15,970	681,106	697,076	44,258,755
Stroke	Stroke	Bothsex	Total	12	1	15,842	842,678	858,520	46,916,547
Gout	Gout	Bothsex	Total	10	1	20,702	823,829	844,531	47,218,578
Chronic obstructive pulmonary disease	COPD	Bothsex	Total	11	1	51,644	750,635	802,279	45,532,064
Asthma	Asthma	Bothsex	Total	13	1	101,311	1,118,682	1,219,993	47,156,680
Idiopathic pulmonary fibrosis	IPF	Female	Total	5	1	1,281	324,340	325,621	30,122,297
Primary open-angle glaucoma	POAG	Female	Total	8	1	3,055	358,334	361,389	30,378,775
Heart failure	HF	Female	Total	6	1	6,414	339,114	345,528	30,796,627
Venous thromboembolism	VTE	Female	Total	4	1	6,210	290,495	296,705	27,492,256
Stroke	Stroke	Female	Total	9	1	4,786	374,631	379,417	31,014,093
Gout	Gout	Female	Total	6	1	2,569	357,103	359,672	31,030,676
Chronic obstructive pulmonary disease	COPD	Female	Total	8	1	16,868	329,201	346,069	29,908,839
Asthma	Asthma	Female	Total	9	1	38,075	339,423	377,498	31,152,977
Idiopathic pulmonary fibrosis	IPF	Male	Total	5	1	1,701	262,944	264,645	28,658,316
Primary open-angle glaucoma	POAG	Male	Total	8	1	2,758	295,037	297,795	29,005,259
Heart failure	HF	Male	Total	6	1	10,288	278,037	288,325	29,315,684
Venous thromboembolism	VTE	Male	Total	4	1	7,080	243,671	250,751	26,399,801
Stroke	Stroke	Male	Total	9	1	5,744	303,338	309,082	29,517,405
Gout	Gout	Male	Total	7	1	9,249	294,636	303,885	29,727,215
Chronic obstructive pulmonary disease	COPD	Male	Total	8	1	18,392	276,146	294,538	29,077,843
Asthma	Asthma	Male	Total	9	1	23,890	284,962	308,852	29,843,298

Table S20. Selection criteria for each analysis included in this study, related to STAR method

Analysis type	Selection criteria
Discovery	MR FDR<0.05, Coloc PP>0.7, Egger intercept P>0.05, Steiger filtering P>0.05
Replication	MR FDR<0.05, Coloc PP>0.7, Egger intercept P>0.05, Steiger filtering P>0.05
Sex-specific analysis	MR FDR<0.05, pair-wise Z-score P males/females <0.05, Egger intercept P>0.05, Steiger filtering P>0.05
Trans-ancestry comparison	MR FDR<0.05 in trans-ancestry comparison, MR P<0.05 in replication, Coloc PP>0.7, Egger intercept P>0.05, Steiger filtering P>0.05
Triangulation	
Robust genetic evidence	MR FDR<0.05 in trans-ancestry comparison, MR FDR<0.05 in replication, Coloc PP>0.7, Egger intercept P>0.05, Steiger filtering P>0.05
Moderate genetic evidence	MR FDR<0.05 in trans-ancestry comparison, MR P<0.05 in replication, Coloc PP>0.7, Egger intercept P>0.05, Steiger filtering P>0.05
Robust observational evidence	Observational association FDR<0.05 and with same direction of effect as MR estimate
Moderate observational evidence	Observational association FDR<0.05 or with same direction of effect as MR estimate
Robust overall evidence	Robust evidence in one type and at least moderate evidence in the other type
Moderate overall evidence	Moderate genetic evidence or observational evidence did not reach the criteria of a moderate evidence level.
